# Association of *TLR2* haplotypes encoding Q650 with reduced susceptibility to ovine Johne’s disease in Turkish sheep

**DOI:** 10.1038/s41598-021-86605-4

**Published:** 2021-03-29

**Authors:** Yalçın Yaman, Ramazan Aymaz, Murat Keleş, Veysel Bay, Cemal Ün, Michael P. Heaton

**Affiliations:** 1 Department of Biometry and Genetics, Sheep Breeding and Research Institute, 10200 Bandırma, Balıkesir Turkey; 2 Department of Biology, Faculty of Science, Ege University, 35000 İzmir, Turkey; 3 USDA, ARS, U.S. Meat Animal Research Center, Clay Center, NE 68933 USA

**Keywords:** Animal breeding, Genetic markers

## Abstract

Ovine Johne’s disease (OJD) is caused by *Mycobacterium avium subsp. paratuberculosis* (MAP) and carries a potential zoonotic risk for humans. Selective breeding strategies for reduced OJD susceptibility would be welcome tools in disease eradication efforts, if available. The Toll-like receptor 2 gene (*TLR2)* plays an important signaling role in immune response to MAP, and missense variants are associated with mycobacterial infections in mammals. Our aim was to identify and evaluate ovine *TLR2* missense variants for association with OJD in Turkish sheep. Eleven *TLR2* missense variants and 17 haplotype configurations were identified in genomic sequences of 221 sheep from 61 globally-distributed breeds. The five most frequent haplotypes were tested for OJD association in 102 matched pairs of infected and uninfected ewes identified in 2257 Turkish sheep. Ewes with one or two copies of *TLR2* haplotypes encoding glutamine (Q) at position 650 (Q650) in the Tir domain were 6.6-fold more likely to be uninfected compared to ewes with arginine (R650) at that position (CI_95_ = 2.6 to 16.9, *p*-value = 3.7 × 10^–6^). The protective *TLR2* Q650 allele was present in at least 25% of breeds tested and thus may facilitate selective breeding for sheep with reduced susceptibility to OJD.

## Introduction

Johne’s disease is a contagious bacterial disease in ruminants caused by *Mycobacterium avium subspecies paratuberculosis* (MAP). It affects cattle, sheep, goats, and wild ruminants world-wide, causing a progressive chronic enteritis resulting in significant production loss. The most common route of disease transmission is fecal–oral, and MAP infections are life-long with no effective treatments or vaccines. Control strategies for Johne’s disease consist of testing for infected animals and removing them from production. However, disease control is hampered by latent MAP infections with long incubation periods and wild-life reservoirs^1,2^. In the sheep industry, annual mortality originated from MAP infection has been estimated at 1 to 4% in New Zealand, 4 to 5% in Cyprus, 6% in the UK, 8 to 12% in Iceland, and 1 to 10% in Australia (reviewed in^3^).

MAP is an obligate intracellular bacterium that is unable to reproduce itself out of the host cells, yet survives for many months in livestock production environments due to its spore-like form. These environments include surfaces, potable water, and some pasteurized conditions^4,5^. Thus, the human food production chain, including powdered infant formulas, can be contaminated with MAP^6^. MAP was ranked second only to coccidiosis as the most important disease among top ten of food-producing animals disease^7^. There is also controversial evidence implicating MAP in the etiology of Crohn’s disease in humans; an inflammatory bowel disease with the relapsing condition in humans^8–10^. MAP can mimic the host cellular components at the molecular level to evade the innate immune system and has been linked to human autoimmune diseases such as Type 1 diabetes, Rheumatoid arthritis, Hashimoto’s thyroiditis, and Multiple Sclerosis due to cross-reactivity between misguided T lymphocytes and host cells (reviewed in^5^). Taken together, MAP infections remain a serious threat to public health, food safety, and animal welfare, and it is important to reduce MAP prevalence by any means possible.

Toll-like receptors (TLRs) are type I transmembrane proteins pattern-recognition receptors (PRRs) that have evolved to detect components of pathogens via pathogen-associated molecular patterns (PAMPs)^11^. Extracellular domains of TLRs are composed of leucine-rich repeats (LRR) that mediate PAMPs recognition, whereas, the intracellular tail of TLRs reacts with an adaptor protein (MyD88) upon activation^12^. Among TLRs, TLR2 has a key role in recognizing the lipopeptide, peptidoglycan, mycolic acids, and lipoarabinomannan PAMPs of gram-positive bacteria and mycobacteria^13–15^. In the search for host alleles influencing susceptibility to Johne’s disease, candidate gene approaches targeting *TLR* gene family have revealed associations in cattle^16–20^ and sheep^21^. Our interest in TLR2 was based on three observations: (1) its increased mRNA abundance soon after MAP infection in sheep^22^, (2) its actively involvement in the innate immune response against tuberculosis^23,24^ and paratuberculosis diseases^16,17^, and (3) its missense variant association with susceptibility to mycobacterial infections in humans and farm animals^25^.

In the present report, our aim was to identify and phase missense variants encoded by *TLR2* in globally-diverse breeds of sheep, and evaluate those present in Turkish sheep for association with OJD. We identified 11 *TLR2* missense variants in whole genome sequence (WGS) data in global populations of 221 sheep representing 61 breeds. PCR-sanger sequencing was used to focus on the four most relevant variants in a Turkish sheep cohort of six native, two crossbred, and three composite breeds from 11 previously described flocks^26^. The study design was a retrospective matched case–control with an indirect-enzyme-linked immunoassay (ELISA) to establish OJD serostatus. PCR-based Sanger sequencing was used to genotype the predominant four missense variants (K137R, D225A, R650Q, F670L) and analyzed by predicted propeptide haplotype with McNemar’s test for association with OJD. Analyses indicated that ewes with one or two copies of the Q650 variant had a 6.6-fold reduced risk for MAP infections. This raises the possibility for use in selective breeding strategies designed to reduce OJD prevalence for disease eradication.

## Results

### OJD seroprevalence of Turkish native and composite sheep, and assembly of matched case–control pairs

Indirect-ELISA results revealed that OJD was wide-spread among all but the most isolated flocks of Turkish sheep. The mean seroprevalence was 7.3% among 2257 ewes from 11 flocks (Tables 1 and S1). The Cine Capari (flock 10) was the only flock free from the MAP infection, and the only flock were reared alone in an isolated upland region. The range of seroprevalence among infected flocks was 1.3% (Karakacan) to 29.4% (Chios) with the two milk breeds (Chios and Awassi) having the highest seroprevalence. From the above 2257 ewes, 120 pairs of seropositive and seronegative animals were identified, where each pair was matched for breed, sex, flock, and age as described in the “Materials and methods”. Of these 120 matched pairs, 18 were eliminated from genetic testing due to difficulties in amplifying their DNA. The remaining 102 ewe pairs (Table 2) were tested for genetic association with *TLR2* missense variants.

**Table 1:** Seroprevalence of OJD.

Breeds	Flock ID	Locations	*n*	OJD serostatus	
				Neg	Pos (%)
K. Merino	1	Bandırma/SRI	901	827	74 (8.2)
Bandirma	2	Bandırma/SRI	366	348	18 (4.9)
Hampshire cross	2	Bandırma/SRI	102	98	4 (3.9)
Ramlic	2	Bandırma/SRI	51	49	2 (3.9)
SBA	2	Bandırma/SRI	36	34	2 (5.6)
Kivircik	2	Bandırma/SRI	208	196	12 (5.8)
Imroz	2	Bandırma/SRI	86	84	2 (2.3)
Chios	3	Bandırma/SRI	51	36	15 (29.4)
Awassi	4	Sanliurfa	36	33	3 (8.3)
Awassi	5	Sanliurfa	35	27	8 (22.9)
Awassi	6	Sanliurfa	43	36	7 (16.3)
Kivircik	7	Kırklareli	50	48	2 (4.0)
Chios	8	İzmir/Cesme	60	46	14 (23.3)
Cine Capari	9	Aydın/Cine	116	116	0 (0.0)
Karakacan	10	Balikesir/Dursunbey	41	40	1 (2.4)
Karakacan	11	Balikesir/Dursunbey	75	74	1 (1.3)
		Total	2257	2092	165 (7.3)

*ID* identifier; *K* Karacabey; *n* number; *OJD* ovine Johne’s disease; *Neg* negative; *Pos* positive; *SRI* Sheep Breeding and Research Institute.

**Table 2:** Distribution of 102 matched case–control pairs.

Breeds^a^	Age of infected ewe										Total pairs
	2	3	4	5	6	7	8	9	11	Un	
K. Merino	7	14	11	13	4	3	–	–	–	–	52
Bandirma	2	–	6	1	1	3	1	–	–	–	14
Kivircik	3	1	2	4	1	–	–	–	–	–	11
Imroz	1	–	–	–	–	1	–	–	–	–	2
Chios	–	3	6	–	–	–	–	1	1	–	11
Karakacan	–	–	–	–	–	–	–	–	–	2	2
Awassi	–	–	–	–	–	–	–	–	–	10	10
Pairs	13	18	25	18	6	7	1	1	1	12	102

*K* Karacabey, *Un* unknown.

### Identification of TLR2 missense variants in WGS from 221 sheep

**Figure 1:**
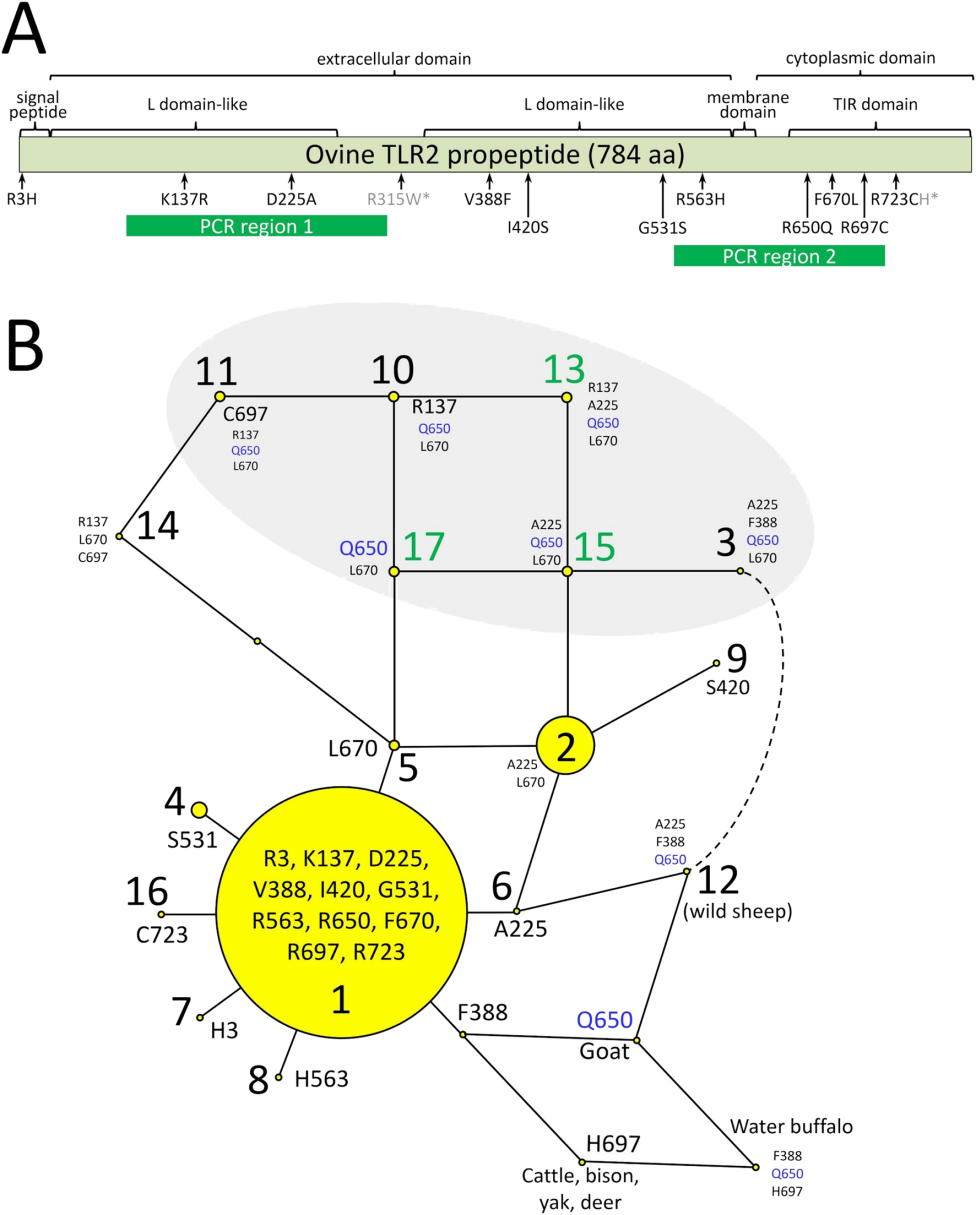
Physical map and phylogenetic tree of phased peptide variants encoded by *TLR2*. (**A**) Sites of amino acid substitution with respect to protein domains. (**B**) Rooted maximum parsimony tree showing relationships of predicted polypeptide isoforms from 171 sheep from 56 breeds. The most frequent TLR2 isoform (“variant 1”) was used as the reference sequence. For “variants 1” through “17”, each node in the tree represents an TLR2 propeptide isoform with one amino acid different compared to adjacent nodes. The areas of the circles are proportional to the variant frequency in the combined group of MSDPv2.4 and ISGC75 sheep panels (n = 171). The grey shaded area encompassed Q650 isoforms in domestic sheep. Loop structures indicate recombination of haplotypes within populations. The TLR2 haplotype nodes in non-*Ovis aries* species were based only on the 11 amino acids positions variant in sheep. The dashed line indicates two species nodes that differ by one variant residue.

The *TLR2* gene is composed of two exons, with the second exon encoding the entire 784 amino acid propeptide sequence. Eleven *TLR2* missense variants were identified in silico by aligning WGS from 221 sheep from 61 breeds around the world (Fig. 1A and Table S2). Haplotype-phased propeptide variants (diplotypes) were unambiguously assigned for animals that were homozygous, or only had one heterozygous missense variant. In the 221 reference animals, 68% of them had unambiguous phased diplotypes (Table S3). Of the remaining 71 ewes with ambiguous diplotypes, 52 (73%) were heterozygous at only two positions and consistent with that of an animal heterozygous for the two most common haplotype alleles (*TLR2* haplotypes “1” and “2”, Fig. 1B). These two haplotype alleles accounted for 83% of the total. The remaining 15 haplotypes were observed with frequencies between 0.03 and 0.003 (Table S4). A comparison of propeptide haplotypes encoded by *TLR2* from closely related bovidae species indicated the ancestral root lies close to haplotypes “1” or “6” (Fig. 1B, Table S5). The presence of loop structures in the ovine *TLR2* phylogenetic tree indicates the occurrence of either recombinant haplotypes or convergent mutations*.* For example, *TLR2* haplotype “2” (A225, L670) has at least three evolutionary paths: meiotic recombination between haplotypes “5” (L670) and “6” (A225), an A225 mutation arising on haplotype “5”, or a L670 mutation arising on haplotype “6”. Although imperfect, organization of the predicted ovine propeptide variants encoded by *TLR2* into a maximum parsimony phylogenetic tree, provides an initial framework for analyzing and interpreting *TLR2* association testing for OJD disease susceptibility.

### Identification of ovine TLR2 missense variants in 102 matched case–control Turkish sheep

**Figure 2:**
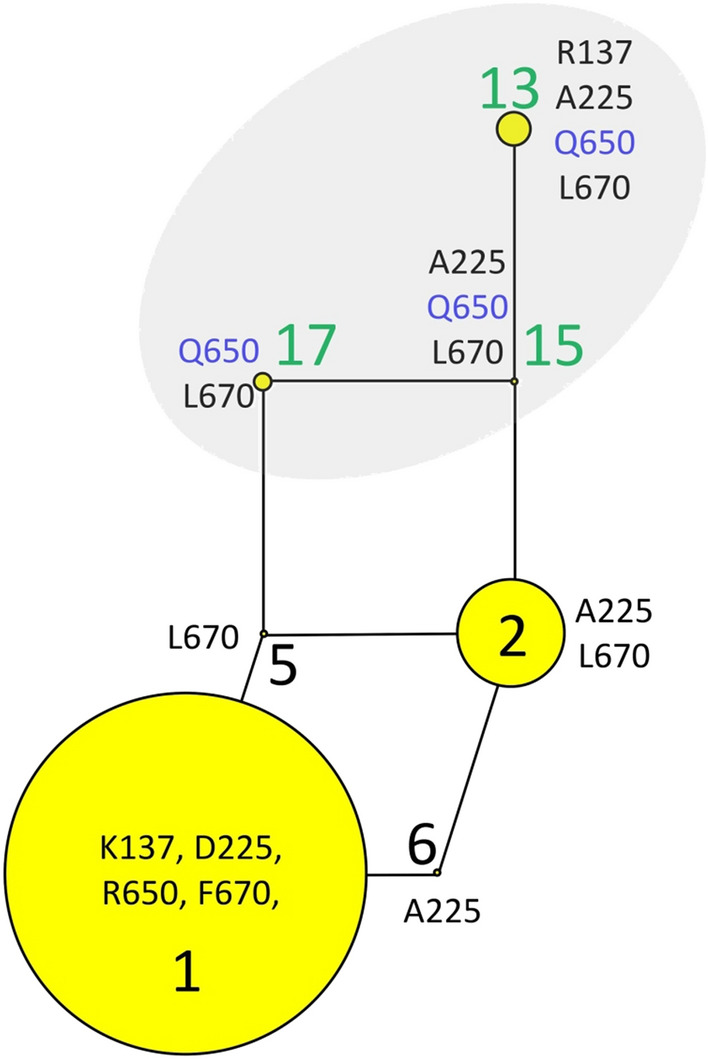
Maximum parsimony tree of polypeptide haplotypes encoded by *TLR2* in the 102 case–control pairs of Turkish ewes. The areas of the circles are proportional to the variant frequency in the 102 matched pairs of ewes. The grey shaded area encompassed Q650 isoforms in Turkish sheep. Loop structures indicate recombination of haplotypes within populations.

Resources were not available for PCR-based Sanger sequencing of the entire propeptide sequence encoded by *TLR2* in the 120 matched pairs of Turkish sheep. Thus, the focus was on two regions containing the most polymorphic missense SNPs: K137R, D225A in the “L domain-like” region, and R650Q, F670L in the Tir domain region (PCR regions 1 and 2, Fig. 1A). Missense SNPs R563H and R697C were contained in these regions, but not polymorphic in the 120 matched pairs. DNA sequencing was successful for 102 of the 120 matched pairs and haplotype assignment was unambiguous for 115 of 204 ewes (56%, Tables S6 and S8). The remaining haplotypes were inferred by using the maximum parsimony tree in Fig. 1 as a framework for interpreting the most probable diplotypes and their frequencies. The resulting maximum parsimony tree for the 102 matched pairs of Turkish sheep was collapsed to seven nodes with propeptide haplotypes “1” and “2” comprising 85% of the total observed (Fig. 2). Based on the frequencies of the propeptide alleles, *TLR2* haplotypes “1”, “2”, and “13” were tested individually as potential risk factors for OJD in 1-copy, 1- or 2-copy, and 2-copy allelic models. Haplotypes “1” and “2” did not reach the target criteria for highly significant results (Methods) in any allelic model, failing on either odds ratio (OR) or number of informative matched pairs (Table 3). Conversely, *TLR2* haplotype “13” with Q650 was strongly associated with significance as a “protective factor” when one copy was present (OR = 0.095, *p*-value = 6.0 × 10^–5^ (Table 3). However, the number of informative pairs for this test was 23, and landed short of the 25 informative pairs recommended for a robust McNemar’s test. Nevertheless, a ewe with one copy of haplotype “13” was 10.5-fold less likely to be infected with MAP than it’s matched seropositive flock mate (CI_95_ 2.4 to 45.4). The effect size of one copy of haplotype “13” was considered large as measured by Cohen’s g (0.41). Since haplotype “13” contained the novel Q650 missense mutation, the association of all haplotypes containing Q650 (i.e., “13”, “15”, and “17”) was also tested as a group (Fig. 2, gray shaded area). When these haplotypes together, the significance of the association increased when one or two copies of any of these Q650-containing haplotypes were present (OR 0.15, *p-*value = 3.7 × 10^–06^, Table 3). This result exceeded the 95% power expectations for detecting an effect with 37% discordant pairs (38/102) with a two-sided McNemar test at a significance level of 0.01. Thus, a ewe with one or two copies of Q650 from derived from any of haplotypes “13”, “15”, and “17”, was 6.7-fold less likely to be infected with MAP than it’s matched seropositive flock mate (CI_95_ 2.6 to 16.9). Like that of *TLR2* haplotype “13” alone, the effect size of one or two copies of Q650 was considered large as measured by Cohen’s g (0.37).

**Table 3:** Association of propeptide haplotypes encoded by *TLR2* with OJD.

TLR2 hap. code	Hap. feature	Risk model	McNemar pairs (case,control)				*b* + *c*	*n*	*(b* + *c)/n*	OR	OR CI_95_		χ^2^	*p*-value (Mid-p)
			+,+	+,−	−,+	−,−								
			*a*	*B*	*c*	*d*					Lower	Upper		
1	Ref. Hap	1 copy	16	21	34	31	55	102	0.54	0.62	0.36	1.06	2.6	4.7 × 10^–2^
1	Ref. Hap	1 or 2 copy	75	16	11	0	27	102	0.26	1.5	0.68	3.13	0.6	1.9 × 10^–1^
1	Ref. Hap	2 copy	17	37	19	29	56	102	0.55	1.9	1.12	3.39	5.2	1.2 × 10^–2^
2	A225, L670	1 copy	11	21	20	50	41	102	0.40	1.1	0.57	1.94	0.0	2.4 × 10^–1^
2	A225, L670	1 or 2 copy	13	27	19	43	46	102	0.45	1.4	0.79	2.56	1.1	1.2 × 10^–1^
2	A225, L670	2 copy	0	8	1	93	9	102	0.09	8.0	1.00	63.96	4.0	3.5 × 10^–2^
13	R137, Q650	1 copy	3	2	21	76	23	102	0.23	0.095	0.02	0.41	14.1	6.0 × 10^–5^
13	R137, Q650	1 or 2 copy	3	2	21	76	23	102	0.23	0.095	0.02	0.41	14.1	6.0 × 10^–5^
13	R137, Q650	2 copy	0	0	0	102	0	102	0.00	na	na	na	na	na
13, 15, 17	Q650	1 copy	3	5	30	64	35	102	0.34	0.17	0.06	0.43	16.5	1.9 × 10^–5^
13, 15, 17	Q650	1 or 2 copy	3	5	33	61	38	102	0.37	0.15	0.06	0.39	19.2	3.7 × 10^–6^
13, 15, 17	Q650	2 copy	0	0	3	99	3	102	0.03	Na	na	na	na	na

“+,+”, “+,−”, “−,+”, and “−,−”; indicate risk factor status within case, control pair; *a, b, c*, and *d*, are McNemar’s quadrants (Methods Table 4).

*CI*_*95*_ 95% confidence interval; *Hap* haplotype; *Ref* reference; *n* number of total pairs; *OR* odds ratio; *χ*^*2*^ the McNemar test statistic with continuity correction.

The distribution of *TLR2* Q650 haplotypes in domestic sheep was limited mostly to breeds native to the Fertile Crescent region. The three*TLR2* Q650 haplotypes associated with OJD in Turkish sheep (“13”, “15”, and “17”) were detected in a total of eight breed groups: Awassi, Bandirma, Chios, Imroz, Karakacan, Kivircik, Karacabey Merino (crossbred of Kivircik), Santa Inês (Table S4). These haplotypes were the most prevalent in native Turkish breeds and these breeds were the likely source of Q650 alleles in improved or composite breeds such as Karacabey Merino, Ramlic, Hampshire crosses, and SBA which were developed backcrossing by European originated terminal breeds. There were three additional *TLR2* Q650 haplotypes (“3”, “10”, and “11”) that were not present in the matched case–control pairs but detected in nine additional breeds: Bangledeshi, Castellana, Changthangi, Dorper, Eastern Tibetan, Garole, Santa Inês, Sumatra, and White Dorper. The effect of these Q650-containing *TLR2* haplotypes is unknown, however it is reasonable to hypothesize they may be associated with reduced susceptibility to OJD.

## Discussion

This report describes the association of *TLR2* haplotypes encoding Q650 with reduced susceptibility to ovine Johne’s disease in Turkish sheep. We combined a candidate gene approach with a retrospective matched case–control design to evaluate propeptide haplotypes encoded by *TLR2* for association with OJD in Turkish sheep. The *TLR2* haplotypes were identified in genomic sequences of 221 reference sheep representing breeds around the world and the 102 matched case–control pairs of Turkish sheep. The 102 pairs were derived from a wide OJD serological survey of 2257 ewes comprising 11 native and composite Turkish breeds. All flocks and breeds tested were infected with MAP, with the exception of one Cine Capari heirloom flock which is isolated from all other sheep and did not have the Q650 allele. Isolation from other infectious sheep is the most likely explanation of the negative MAP status of the Cine Capari and the purpose of this flock’s isolation. The vast majority of seropositive ewes in this study appeared to be clinically normal which is typical of MAP infections. The serological results suggest that all but the most isolated Turkish flocks are infected with MAP and at risk for OJD. This is consistent with other reports of OJD prevalence around the world^27–29^.

A critical step in evaluating candidate genes for association with traits is defining the spectrum of protein variants^30,31^. Gene function in livestock may be affected by a wide range of large and small scale genomic sequence differences^32,33^. However, variants that alter amino acid sequences via missense, nonsense, frameshift, and splice site variants, are readily detectable in sheep WGS and among those most likely to affect function^34^. Thus, defining the prevalence and diversity of the predicted propeptide haplotypes encoded by *TLR* in global sheep populations was important for understanding function and identifying which breeds are affected. This report identified 11 missense variants comprising 17 predicted propeptide haplotypes encoded by *TLR2* in 221 sheep from 61 breeds from around the world and in Turkish sheep. Frameshift, splice site, and nonsense mutations were not detected in this study, nor were the R315W or the R723H variants reported in dorper sheep^35^. Organizing the phased propeptides encoded by *TLR2* into a rooted phylogenetic tree in global sheep populations was important for inferring phased diplotypes in Turkish sheep and gaining insight into the evolutionary history of the coding sequence. The phylogenetic tree structure had multiple loops involving infrequent haplotypes and was suggestive of recombination within the *TLR2* exon 2 and consistent with directional selection away from the root, and towards more recent haplotypes.

A retrospective matched case–control design, combined with McNemar’s test for correlated proportions, has been a successful approach for genetic association studies with other chronic infectious diseases in sheep^26,36^. The pairwise identification of affected and unaffected sheep matched for age, year, sex, breed, flock, and location appears to effectively control for confounding factors like pathogen exposure, pathogen strain, exposure duration, and admixture. The present study had good statistical power to evaluate the three most frequent *TLR2* propeptide haplotypes in 102 matched pairs of Turkish sheep (haplotypes “1”, “2”, and “13”, Fig. 2). Only *TLR2* haplotype “13”, with its Q650 variant was significantly associated with OJD. This observation was reinforced in combined analyses with the other *TLR2* haplotype harboring the Q650 variant (haplotypes “15” and “17”). This suggests that selective breeding for *TLR2* haplotypes “13”, “15” and “17” in Turkish flocks may reduce the genetic susceptibility of animals to OJD. Moreover, increasing the frequency of *TLR2* Q650 variants in Turkish flocks may reduce the incidence of OJD in affected flocks.

The impact of the Q650 variant on the TLR2 protein function is unknown. The missense variant is located in the cytoplasmic TIR domain and reduces the net negative charge by one compared to the R650 residue. TIR domain interactions between host cellular receptors and adaptors are important for activating conserved cellular signal transduction pathways in response to bacterial and viral pathogens, cytokines and host growth factors^37^. However, it is also possible that the Q650 variant is linked to a nearby, causal mutation that does not affect the primary sequence of the TLR2 protein. There are a number of related species that suffer from Johne’s disease, yet have Q650 as their predominant residue at that position, including bighorn sheep^38^, goats, and water buffalo^39^ (Table S7). Moreover, sequencing the TIR region of *TLR2* in 14 saanen goats with OJD showed all were homozygous Q650 (data not shown). The most recent common ancestor (MRCA) between goats and sheep is approximately 10 million years ago^40^ and thus the Q650 allele may be rather ancient. Species alignments with TLR2 propeptide sequences show the Q650 residue present in ruminants, cetacea, ungulates, primates, rodents, amphibians and jawless vertebrates such as lamprey. The latter shares a MRCA approximately 615 million years ago^40^ with sheep (Tables S5 and S7). Furthermore, the *TLR2* Q650 variant is present in the human 1000 genomes data set with < 0.01 frequency (rs200483398) and listed as “neutral” and “well tolerated”^41^. Thus, if the presence of the ovine Q650 residue is responsible for the association of *TLR2* with OJD in Turkish sheep, perhaps it is also dependent on a limited number of combinations of the other 783 residues encoded by *TLR2*.

The occurrence of Q650 variants was previously reported in Djallonke, Dorset, and Red Maasai^35^, however their frequencies and phased diplotypes were not provided for these sheep, and thus could not be compared here. Although, breeds like Bangledeshi, Castellana, Changthangi, Dorper, Eastern Tibetan, Garole, Santa Inês, Sumatra, and, White Dorper may provide a potential source for *TLR2* Q650 alleles, it is unknown whether haplotypes “13”, “15”, and “17” are associated with reduced susceptibility to OJD in those breeds, and whether other haplotypes containing Q650 (e.g., “3”, “10”, and “11”) are also associated with infection. Thus, more studies are needed to determine whether the results observed in the present report may be extended to other populations and production environments.

In conclusion, *TLR2* haplotypes encoding Q650 were associated with reduced susceptibility to ovine Johne’s disease in Turkish sheep. Ewes with one or two copies of the Q650 variant on haplotypes “13”, “15”, and “17” had a 6.6-fold reduced risk for MAP infections. This suggests that selection for *TLR2* Q650 alleles in Turkish sheep may be useful for reducing OJD prevalence in Turkish sheep. Moreover, it raises the possibility that *TLR2* haplotypes encoding Q650 in other breeds or species may affect susceptibility to MAP infections and Johne’s disease. However, further research is needed to replicate the present results in other affected flocks segregating these *TLR2* haplotypes encoding Q650 alleles.

## Methods

### Animals

For OJD testing and association, this study included six native (Kivircik, Imroz, Chios, Awassi, Cine capari, and Karakacan), two cross-bred (Kivircik x Merino [Karacabey Merino] and Rambouillet x Daglic [Ramlic]), and three composite (Bandirma, Hampshire crosses, and Black head merino crosses) Turkish sheep from eleven flocks (*n* = 2257). To provide sufficient time for ewe exposure and seroconversion, only animals two years-old and older were included for this study. Most of the ewes were from three research flocks at Sheep Breeding and Research Institute (SRI) and others were from farmers’ flocks. The composite sheep, Hampshire crosses, Black Head Merino crosses (SBA), and Bandirma were developed at SRI by crossing native breeds with terminal rams originating from Europe to improve meat yield of native sheep. Among native breeds, Kivircik is renowned for its meat flavor whereas Awassi, Chios and Imroz are known for their high milk yield. Karacabey Merino and Ramlic sheep were improved in the 1940′s and closed the backcrossing for at least the last 30 years. With their population size now less than 200 adult ewes, the Cine Capari and Karakacan breeds are in danger of extinction.

For *TLR2* missense variant identification in global sheep populations, this study used WGS aligned to NCBI reference genome Oarv3.1 from three collections of animals: (1) the US Meat Animal Research Center (USMARC) Sheep Diversity Panel version 2.4 (MSDPv2.4, BioProject PRJNA324837) containing 96-animals and 11 breeds^34^; (2) the USMARC Extended Sheep Diversity Panel version 1.0 (MESDPv1.0) containing 50-animals and 5-breeds (unpublished); and 3) the International Sheep Genome Consortium (ISGC75) containing 75-animal, 43 breeds, and 2 wild species^42^. The average read depth over all 221 animals was approximately 15-fold with a minimum of ninefold and a maximum of 34-fold. Fourteen Johne’s positive Saanen goats from a small flock of SRI goats were also used to evaluate the TLR2 residue encoded at position 650. These infected goats ranged in age from two to six years.

### Sample collection and Serological analysis

Peripheral whole blood with and without EDTA was sampled from all individuals by venipuncture. Serum was obtained by centrifugation of fresh whole blood without EDTA and stored at – 20 °C until use. An indirect-ELISA was used to detect serostatus of 2257 samples per the manufacturer’s instructions (Idexx Laboratories, inc. Westbrook, USA). After incubation and washing protocols, ELISA plates were read at 450 nm wavelength.

### Pairwise ewe matching

Controlling for pathogen exposure is essential for successful genetic association studies involving susceptibility to infectious disease. Retrospective matched case–control designs can help control for exposure intensity and duration to persistent infectious diseases like OJD. MAP exposure was controlled by using ewes older that two years and matching for sex, age, breed, flock, and location. This also accounted for possible population stratification. Where possible, the age criterion included having the seronegative ewe be the same age or older than the seropositive pair mate to allow for equivalent or increased exposure. Thus, the aim was to match a seropositive ewe with a seronegative ewe from the same flock, same breed type, and same or older age (Table S9). Ewe age was not available for some private flocks, and thus approximately 10% of the pairs were not ideally matched for MAP exposure duration.

### PCR-based Sanger sequence genotyping

To investigate variable regions of the ovine *TLR2 coding sequence*, two separate PCR was carried out using the designed primers; TLR2_F_1: GGGGGCCAATGAAATTCACAC, TLR2_R_1: GTCAGTGCTGTAAAATCGCCA and TLR2-F_2: ACCACTCGCTCCTCACAAAG, TLR2-R_2: GACTTCCTGTCCTTCGCACA, thus, 1168 bp of the 2355 bp coding region of ovine *TLR2* was amplified in total. The TIR region of caprine *TLR2* was amplified with the same primers. PCR products were sequenced with following protocols; exo-sap incubation, chain termination, ethanol purification, and capillary electrophoresis (Applied Biosystems ABI 3500).

### Assigning haplotype phase and assembling a phylogenetic tree

Haplotype-phased propeptide variants predicted to be encoded by *TLR2* were unambiguously assigned in individuals that were either: (1) homozygous for all 11 missense variants, or (2) had exactly one heterozygous missense variant. A maximum parsimony phylogenetic tree was manually constructed from the unambiguously phased protein variants. This tree was used, together with simple maximum parsimony assumptions, to infer *TLR2* haplotype phase in ewes where two or more heterozygous missense variant sites occurred in. The protein phylogenetic trees were rooted by comparing the 11 missense variant sites in sheep to the equivalent 11 sites in other species, starting with those most closely related. The ovine peptide sequence encoded by *TLR2* was used to search NCBI's refseq_protein database with BLASTP (version 2.6.123^43^). Aligned protein sequences from a representative subset of 33 species throughout the vertebrata subphylum were used for the comparison.

### McNemar’s test for correlated proportions

Propeptide isoforms predicted to be encoded by *TLR2* (i.e., haplotype alleles) were tested as possible disease risk factors in three different models: 1-copy, 1- or 2-copy, and 2-copy settings. The presence (+) or absence (−) of a possible genetic risk factor was assigned to each animal and the matched pair was then classified by one of four possible binomial outcomes (Table 4).

**Table 4:** McNemar pair classifications in a 2 × 2 contingency table.

		Unaffected		Row totals
		Genetic risk factor present (case,control)	Genetic risk factor absent (case,control)	
Affected	Genetic risk factor present	+ , + (*a*)	+ , − (*b*)	*a* + *b*
	Genetic risk factor absent	−, + (*c*)	−, − (*d*)	*c* + *d*
		*a* + *c*	*b* + *d*	*n*

In McNemar’s test, only the discordant pairs are informative (i.e., quadrants b and c). A genetic risk allele was assigned when quadrant b was greater than quadrant c (i.e., OR > 0). A protective allele was assigned when quadrant c was greater than quadrant b (i.e., OR < 0). A power analysis was performed using G*Power v3.1.9.4 software^44^ to evaluate the statistical strength of the study. The target criteria for highly significant results were an OR greater than or equal to 3 and alpha of 0.05, and at least 25 total informative pairs (i.e., (b + c)/n). Although not all haplotypes tested had 25 informative pairs, the mid-*p* was used since it performs well in these situations compared with the asymptotic, asymptotic with continuity correction, and exact conditional tests^45^. Cohen’s *P* (c/[b + c]) and Cohen’s g (Cohen’s *P*—0.5) were used to roughly estimate the effect size^46^.

### Ethics declarations

All animal procedures in the study were reviewed and approved by the ethics committee of Sheep Breeding and Research Institute (Approval Number: 1282412), and the authors complied with the ARRIVE guidelines.

## Supplementary Information

Supplementary Table S1.

Supplementary Table S2.

Supplementary Table S3.

Supplementary Table S4.

Supplementary Table S5.

Supplementary Table S6.

Supplementary Table S7.

Supplementary Table S8.

Supplementary Table S9.
